# When Two Syndromes Collide: Managing Fanconi and Refeeding Syndrome in a Single Patient

**DOI:** 10.7759/cureus.52169

**Published:** 2024-01-12

**Authors:** Francisco J Gallegos Koyner, Nelson Barrera, Prakriti Subedi, Karun Shrestha, Roberto Cerrud-Rodriguez

**Affiliations:** 1 Internal Medicine, St. Barnabas Hospital Health System, New York City, USA; 2 Cardiovascular Medicine, Yale New Haven Hospital, New Haven, USA

**Keywords:** recurrent hypoglycemia, infectious esophagitis, upper gastro-intestinal bleed, proximal renal tubule, fanconi, refeeding, tenofovir alafenamide (taf), hiv aids

## Abstract

Refeeding syndrome is the potentially fatal shift in fluids and electrolytes that may occur in malnourished patients after receiving artificial refeeding. Its hallmark feature is hypophosphatemia, although other electrolytes might also be affected. Fanconi syndrome is a generalized dysfunction of the proximal tubule characterized by proximal renal tubular acidosis (RTA), phosphaturia, glycosuria, aminoaciduria, and proteinuria. The etiology of Fanconi syndrome can be either acquired or inherited, and drugs, among them tenofovir, are a common acquired cause of this disease. We present the case of a patient with AIDS and polysubstance abuse who was admitted due to pneumonia, completed treatment, was then started on antiretroviral medication (ART) that included tenofovir alafenamide (TAF) and began presenting severe episodes of hypophosphatemia along with other electrolyte imbalances, leading the workup denoted in the case, severe complications and finally to the patient's demise. Most cases of tenofovir-related Fanconi syndrome are related to tenofovir disoproxil fumarate, but very few cases have been reported with TAF. Our case highlights this rare complication of therapy with TAF and how artificial feeding can contribute to severe electrolyte abnormalities and worsen outcomes.

## Introduction

Fanconi syndrome, or proximal renal tubular dysfunction, is a syndrome characterized by proximal renal tubular acidosis (RTA), phosphaturia, renal glucosuria, and others. While Fanconi syndrome has been primarily associated with tenofovir disoproxil fumarate (TDF), its occurrence in patients receiving tenofovir alafenamide (TAF) remains relatively rare. Here, we present a patient who developed Fanconi syndrome following TAF exposure, for which the drug had to be discontinued. 

Furthermore, we wish to highlight the confluence of Fanconi syndrome and refeeding syndrome in our case, an intricate interplay that can lead to severe complications and even fatal outcomes. Malnourished patients, when subjected to artificial nutrition, are at risk of developing refeeding syndrome, characterized by a wide array of electrolyte abnormalities. Our case illustrates the complex challenges faced in the clinical management of these two syndromes and how they can lead to fatal outcomes.

This article was previously presented as a meeting abstract at the 2023 CHEST Annual Meeting on October 9, 2023. 

## Case presentation

We present the case of a 57-year-old female with HIV/AIDS not taking her HIV medications and polysubstance use, specifically crack cocaine and alcohol, who presented to the emergency department (ED) complaining of shortness of breath, cough and yellow sputum for a few months, along with epigastric pain and non-radiating chest pain which worsened with cough. The patient had prior hospitalizations for similar symptoms but left against medical advice multiple times. On review of systems, the patient acknowledged night sweats, weight loss, generalized weakness, pleuritic chest pain, dyspnea on exertion, dysphagia, and odynophagia.

**Figure 1:**
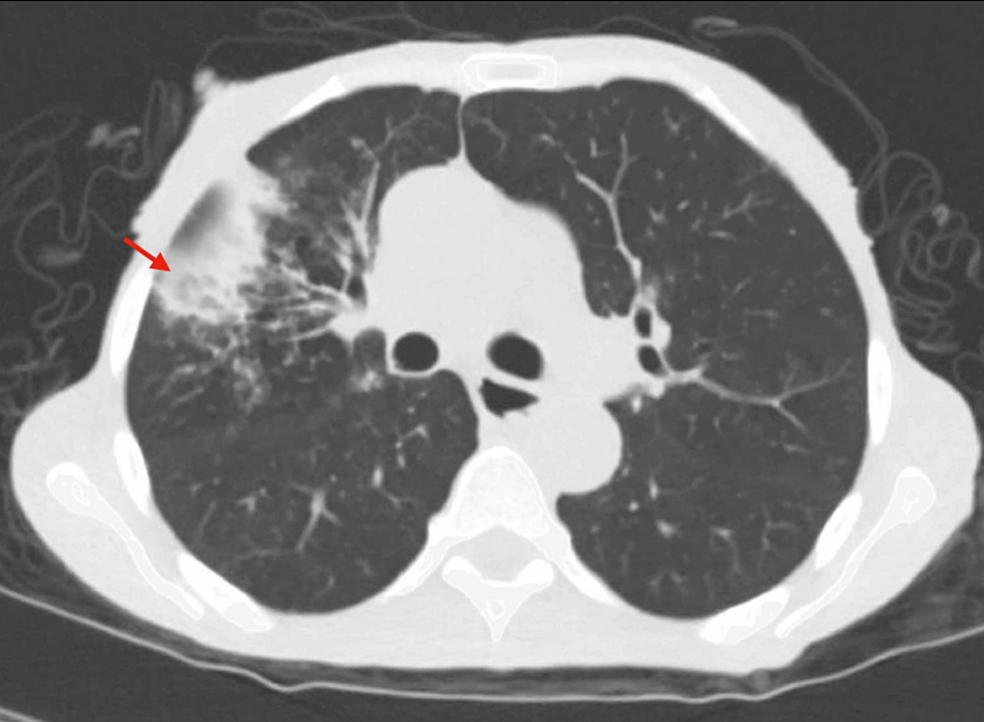
CT chest without contrast. CT was reported as interval development of branching opacities (tree-in-bud) and pleural-based areas of consolidation in the right upper and lower lobe, suggestive of pneumonia superimposed on chronic interstitial changes (as signaled by the red arrow). Additionally, these findings were also described: interval development of thickening of the minor fissure on the right. Diffuse bronchiectasis. Emphysema. Interval improvement of pleural-based opacity at the medial basal segment of the left lower lobe compared to previous imaging. Interval improvement of pleural-based opacity in the posterior basal segment of the right lower lobe compared to previous imaging.

Initial vital signs were blood pressure of 118/78 mmHg, heart rate of 64 bpm, temperature of 97.2ºF, and oxygen saturation of 98% on room air. The physical exam was positive for oral thrush and epigastric tenderness. The chest x-ray showed right lung opacities, which were interpreted as "most likely early multifocal pneumonia." CT chest without contrast reported "interval development of branching opacities (tree-in-bud) and pleural-based areas of consolidation in the right upper and lower lobe suggestive of pneumonia superimposed on chronic interstitial changes" (Figure 1). Emphysematous changes and diffuse bronchiectasis were also seen.

Laboratories were remarkable for normocytic anemia with hemoglobin of 8.9 g/dL, CD4 count of 8 cells/µL, acid-fast bacilli (AFBs) and polymerase chain reaction (PCR) for *Mycobacterium tuberculosis* were negative, serum 1,3-β-D-glucan (Fungitell) was normal, influenza A was positive and procalcitonin was elevated. No leucocytosis or fever was recorded. 

**Figure 2:**
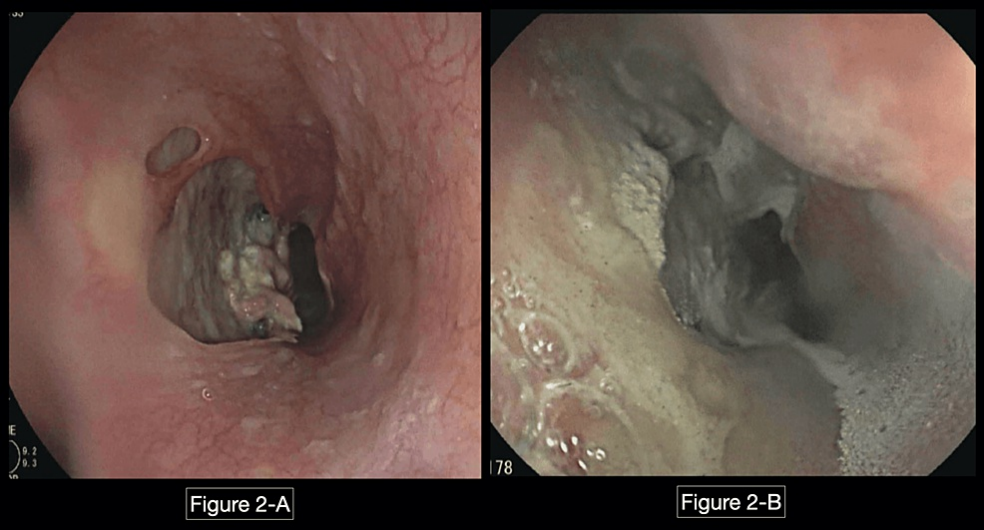
EGD showing esophageal ulcers. Image in the left (A) was taken at the first EGD at the level of the middle esophagus, where an esophageal ulcer can be appreciated, which was biopsied. Image in the right (B) was taken during the second EGD, which was done emergently due to gastrointestinal bleeding, but at the same time, it was done after completing fluconazole therapy. The patient was still complaining of severe dysphagia and odynophagia at the time of the second EGD. EGD: esophagogastroduodenoscopy.

The patient was started on treatment for influenza with superimposed bacterial pneumonia and was being empirically treated for *Candida esophagitis* with fluconazole. Since there was no improvement in her dysphagia after several days of empiric treatment, esophagogastroduodenoscopy (EGD) was performed, which showed esophageal ulcers and an ulcerated lesion, both biopsied (Figure 2). Biopsy showed: "scant cellular debris, unable to characterize due to limited size of the sample. No viral cytopathic effect is present in the submitted material. Single minute fragment of fibrous tissue with acute and chronic inflammatory aggregates, consistent with ulcer bed." Additionally, the fundoscopic eye exam did not show evidence of cytomegalovirus (CMV), and the endoscopic biopsy was negative for CMV, but the sample size was very small and a repeat endoscopy was needed. 

Respiratory symptoms improved and antibiotics were completed for seven days, and then bictegravir, emtricitabine, and tenofovir alafenamide (B/F/TAF) was started in the patient. Soon after initiation of B/F/TAF, the patient started having multiple episodes of hypoglycemia, for which dextrose 5% in water (D5W) had to be started. Initially, hypoglycemia was attributed to starvation as the patient was not eating due to dysphagia, but due to the severity of the episodes (capillary blood glucose (CBG) <20) further explanation was needed. A urinalysis was taken and showed 150 mg/dL of glucose, despite the patient never being hyperglycemic, so Fanconi syndrome was suspected. Fanconi syndrome was confirmed by a paradoxically high urinary phosphorus level in a patient with hypophosphatemia: fractional excretion of phosphate was 67% (below 20% is considered normal). B/F/TAF was immediately discontinued. 

The patient persisted with severe dysphagia and was unable to tolerate oral feedings; therefore, in a joint decision with her, the decision to place a percutaneous gastrostomy (PEG) tube was made, and enteral feeding was started as per nutritional recommendations with hopes of weaning off D5W.

**Figure 3:**
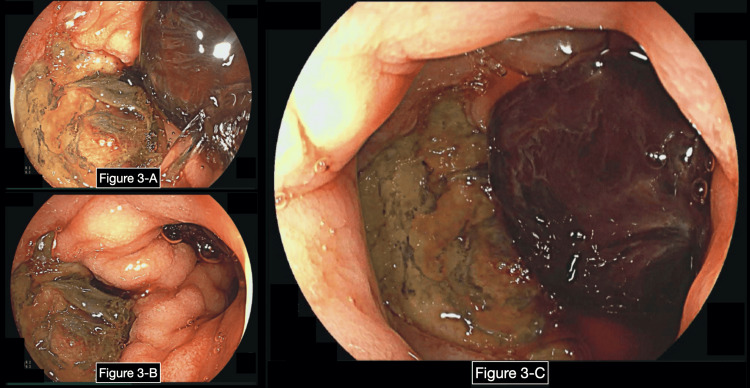
Second EGD showing a duodenal bulb ulcer. These are images taken from the second EGD after a concern for upper GI bleeding developed. Figures (A)-(C) are images taken from the duodenal bulb. (C) shows the duodenal bulb where a duodenal ulcer with a visible vessel (Forrest IIa) was found. EGD: esophagogastroduodenoscopy.

A few days later, the patient developed melena; therefore, enteral feedings were stopped, pantoprazole was started, and D5W was switched to dextrose 10% in water (D10W). Hemoglobin decreased the same day from 8.5 g/dL to 5.7 g/dL, and the patient required multiple blood transfusions. EGD was repeated urgently, hemostatic spray was applied to esophageal ulcers and a duodenal ulcer Forrest IIa (Figure 3). That same night, partial parenteral nutrition (PPN) was started. 

**Figure 4:**
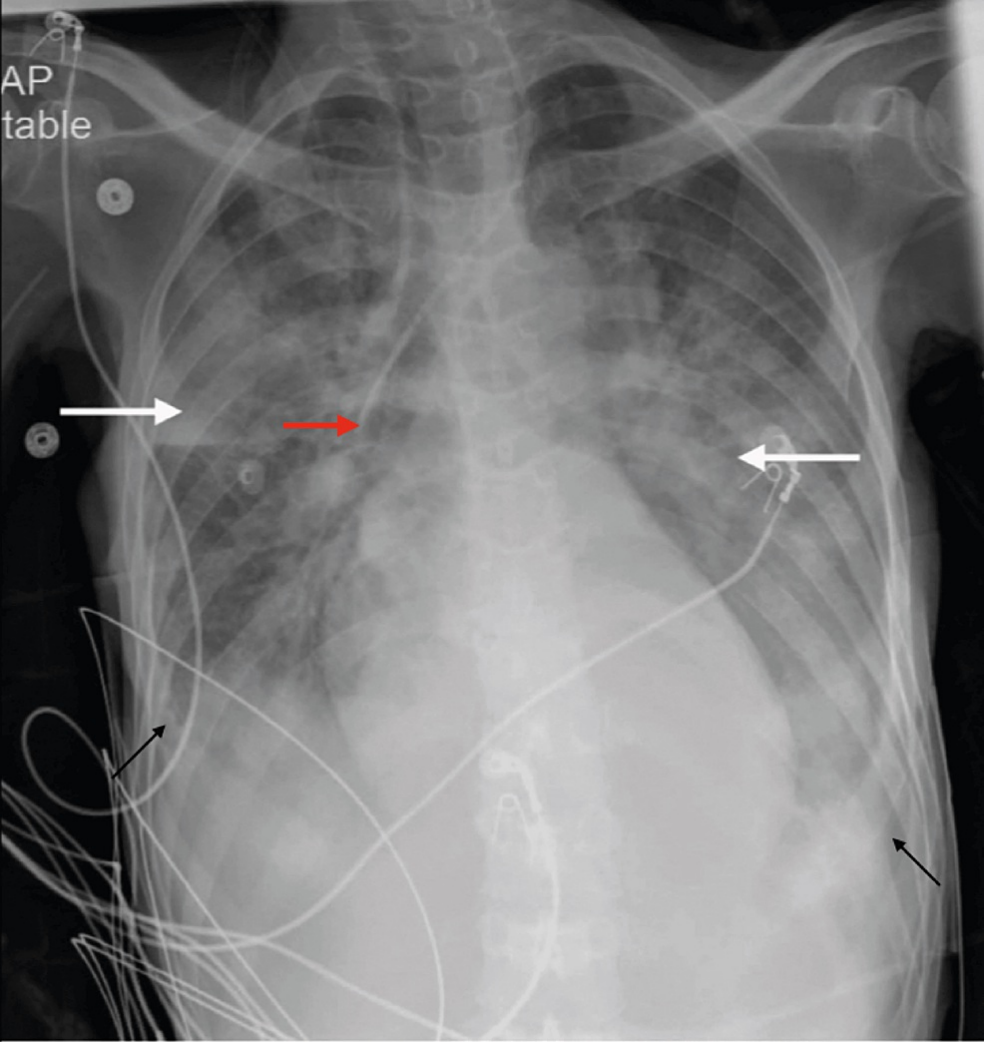
Chest x-ray taken in the ICU showing bilateral pulmonary edema. Chest x-ray was taken in the intensive care unit and read as follows: a left IJ central line catheter is placed, terminating in the projection of mid-SVC (red arrow). Hyperinflated lungs are seen. Widespread patchy alveolar interstitial ground-glass opacities in both lung fields are seen (both white arrows). Likely small bilateral pleural effusion present (black arrows). SVC: superior vena cava.

By the next morning, the patient had received approximately 300 g of dextrose between the combination of D10W and PPN and presented severe refeeding syndrome with severe hypophosphatemia, hypokalemia, and hypomagnesemia. She was transferred to the intensive care unit (ICU) for central line placement and aggressive repletion of these electrolytes. Subsequently, she had an increase in her oxygen requirements, from two liters nasal cannula to 15 liters non-rebreather mask. Chest x-ray then showed bilateral patchy opacities consistent with pulmonary edema (Figure 4), which could be attributed to the severe refeeding syndrome. Despite further measures, the patient continued to decompensate and died a few days later. 

## Discussion

Fanconi syndrome is a rare renal disorder characterized by impaired reabsorption of solutes in the proximal renal tubule, leading to excessive urinary losses of substances such as glucose, amino acids, phosphate, uric acid, and bicarbonate [1]. This syndrome can be inherited or acquired. Primary inherited Fanconi syndrome is most commonly due to a mutation in the sodium-phosphate cotransporter (NaPi-II) in the proximal tubule [2], although other genes have also been associated [3,4]. Fanconi syndrome can also be associated with inherited systemic diseases, of which the most common is cystinosis [5], but other associations include Dent disease, Lowe disease [6], galactosemia, hereditary fructose intolerance, tyrosinemia, Alport syndrome, Wilson disease, and mitochondrial disorders.

On the other hand, acquired causes of Fanconi syndrome are most commonly seen with drug-induced nephrotoxicity, followed by light-chain-associated Fanconi syndrome [1,7]. Drugs that have been linked to acquired Fanconi syndrome include antiretroviral medications (ARTs), such as tenofovir [1,8,9], adefovir, cidofovir, drugs such as ifosfamide [10], oxaliplatin [11], cisplatin [12], and anticonvulsants such as topiramate and valproic acid [13]. Tenofovir disoproxil fumarate has been widely linked to Fanconi syndrome, and due to its severe nephrotoxicity, it has been widely replaced with tenofovir alafenamide, which has also been linked to Fanconi syndrome in two case reports [1,14,15].

In our case, the development of Fanconi syndrome was likely linked to the initiation of TAF, which is part of the combination B/F/TAF, a commonly used ART for patients with HIV. Discontinuation of the offending medication, in this case B/F/TAF, is a crucial step in managing Fanconi syndrome, followed by supportive care, which involves addressing electrolyte imbalances. Renal dysfunction is usually reversible with the cessation of the drug. 

Refeeding syndrome is a complex and potentially life-threatening condition characterized by a shift in fluids and electrolytes that occurs in malnourished individuals when there is a rapid reintroduction of nutrition, especially carbohydrates [16,17]. Refeeding syndrome is characterized by electrolyte abnormalities, and its hallmark feature is hypophosphatemia [16], but other abnormalities such as hypokalemia, hypomagnesemia, thiamine deficiency, sodium abnormalities, and among others, may also develop. Our case highlights the development of refeeding syndrome in a patient who required artificial feeding due to gastrointestinal bleeding.

Prevention of refeeding syndrome involves, first of all, identifying high-risk patients, checking baseline electrolyte levels and repleting them, administering thiamine and multivitamins prior to artificial feeding, and then starting feedings with a gradual increase in calorie intake, close monitoring of electrolyte levels, and prompt correction of imbalances. Management of refeeding syndrome involves identifying it and repletion of electrolytes, of which the most common is phosphorus [16,17]. In severe cases, as seen in this patient, intensive care management might be required. 

The collision of Fanconi syndrome and refeeding syndrome in the same patient underscores the complexity of managing such cases and highlights the importance of differentials to include in a patient who develops hypophosphatemia during their hospitalization. This case also serves as an example of how a rare differential can be brought only by the interpretation of urinalysis in the right clinical context-this being a patient with glycosuria in the context of repeated episodes of hypoglycemia, raising the suspicion that the proximal renal tubule is not functioning adequately. Finally, the fact that Fanconi syndrome developed after the use of TAF is something poorly reported in the literature [14,15]. 

## Conclusions

The development of Fanconi syndrome following the initiation of TAF underscores the need for vigilant monitoring of patients on ART, as renal complications can manifest even with newer, safer medications. The importance of recognizing and promptly discontinuing medications causing adverse effects cannot be overstated. In addition to this, while managing complications such as recurrent hypoglycemia and electrolyte abnormalities resulting from Fanconi syndrome, physicians must always remind themselves of the risk of refeeding syndrome and work with nutritionists to prevent its development. 

The tragic outcome of this case serves as a somber reminder of the critical importance of comprehensive care, close monitoring, and prompt intervention in the management of malnourished patients with HIV/AIDS, shedding light on the challenges and opportunities for advancing patient care in this context. 
